# Predicting schizophrenia spectrum disorders in pediatric outpatients: a prospective validation of the child psychosis-risk screening system

**DOI:** 10.3389/frcha.2026.1840330

**Published:** 2026-06-17

**Authors:** Yukiko Hamasaki, Shugo Michikoshi, Yuko Sakaue, Takao Nakayama, Satoko Ueba, Naoki Kurimoto, Yoshiteru Mutsuda, Masanori Isobe, Toshiya Murai, Takatoshi Hikida

**Affiliations:** 1 Faculty of Contemporary Society, Kyoto Women’s University, Kyoto, Japan; 2 Department of Psychiatry, Shigasato Hospital, Otsu, Japan; 3 Faculty of Data Science, Kyoto Women’s University, Kyoto, Japan; 4 Department of Pediatrics, Shiga University of Medical Science, Otsu, Japan; 5 Department of Pediatrics, Saiseikai Moriyama Municipal Hospital, Shiga, Japan; 6 Department of Psychiatry, Shiga University of Medical Science, Otsu, Japan; 7 Department of Psychiatry, Kyoto University Graduate School of Medicine, Kyoto, Japan; 8 Laboratory for Advanced Brain Functions, Institute for Protein Research, The University of Osaka, Osaka, Japan

**Keywords:** child behavior checklist, child psychosis risk, developmental psychopathology, early detection, longitudinal prediction, primary care screening, schizophrenia spectrum disorders, screening tools

## Abstract

**Objective:**

This study validated the prognostic ability of the Child Psychosis-risk Screening System (CPSS), an algorithm derived from the widely used Child Behavior Checklist (CBCL), and benchmarked its performance against a comprehensive machine learning model.

**Methods:**

This prospective cohort study enrolled 491 pediatric psychiatric outpatients aged 6–18 years who visited clinics for developmental, psychosomatic, or psychiatric disorders. Baseline assessments (Visit 1) included the CBCL and extensive clinical data. Participants were followed-up for up to 5 years (n = 350; Visits 2+), including annual assessments for schizophrenia spectrum disorders (SSD). The predictive accuracy of the CPSS risk score was evaluated using receiver operating characteristic analysis and compared with that of a post-hoc machine learning benchmark model (Light Gradient Boosting Machine) incorporating 150 baseline variables.

**Results:**

The CPSS risk score demonstrated good predictive performance for final SSD status (area under the curve [AUC] = 0.906), significantly exceeding its discriminative ability for SSD at Visit 1 (AUC = 0.841). This discrepancy indicates that the CPSS sensitively captures prodromal vulnerabilities that do not yet meet diagnostic criteria. Although the complex Light Gradient Boosting Machine model achieved a marginally higher AUC (0.959), the CPSS yielded comparably robust accuracy using only eight CBCL subscales. Variable-importance analysis further showed that the CPSS risk score was a stronger predictor than the CBCL “Thought Problems” scale, a traditional marker of psychosis risk.

**Conclusion:**

The CPSS is a parsimonious and effective screening tool that translates behavioral observations into objective risk stratification. Its use in primary care settings could streamline referral pathways and facilitate timely early intervention for children at high risk of psychosis who present with ambiguous somatic or behavioral symptoms.

## 1 Introduction

Schizophrenia is a severe psychiatric disorder affecting approximately 0.75% of the global population (1, 2). The condition not only profoundly impacts affected individuals but also represents a significant loss of human capital, with its economic burden estimated to range from 0.02% to 1.65% of the gross domestic product across different countries (3, 4). In recent years, increasing emphasis has been placed on early detection and intervention, as evidence indicates that a longer duration of untreated psychosis (DUP) is associated with poorer outcomes (5, 6), whereas reducing the DUP contributes to symptomatic and functional improvements (7). This has driven the development of tools to prospectively identify and monitor individuals with a high risk for psychosis based on clinical and genetic risk factors (8–11).

Early-onset schizophrenia (EOS; onset in childhood or adolescence) is associated with particularly severe symptoms and poor prognosis (12–14). Meta-analyses have shown that EOS has a higher genetic and neurodevelopmental burden and a higher likelihood of residual negative symptoms and cognitive decline than adult-onset schizophrenia (15). Furthermore, because it occurs during a critical period of identity formation, education, and socialization, it is more likely lead to poorer social outcomes (e.g., academic interruption and employment difficulties) than adult-onset schizophrenia (16–18). In other words, in addition to the severity of the symptoms themselves, patients with EOS also have a secondary poor prognosis (poor social functioning) due to impaired achievement of developmental goals. Treatment after onset alone is not sufficient to improve outcomes. Consequently, early detection and intervention at the prodromal/at-risk mental state stage are crucial for changing this “poor prognosis trajectory”.

The diverse and nonspecific nature of early-stage psychiatric symptoms in children presents a considerable challenge for objective assessments (19). During the premorbid and prodromal phases, nonspecific behavioral changes are often prominent, such as developmental delays, social withdrawal, and school refusal (20–23). Typically, it is only after the emergence of specific psychotic symptoms that these children receive attention from psychiatric professionals (24, 25).

Notably, children in the premorbid and prodromal phases frequently present with somatic complaints alongside behavioral problems, a characteristic feature of this age group (26, 27). Indeed, it is not uncommon for adolescents experiencing their first episode of psychosis to have a prolonged history of treatment in pediatric or internal medicine settings prior to their first psychiatric consultation. The period preceding psychiatric referral represents a crucial window of opportunity for early identification of children at risk for psychosis. Implementing an accurate screening method at this stage could facilitate a smoother transition to psychiatric care and enable timely preventive interventions.

Currently, research on the clinical high-risk for psychosis state in children remains in its early stages, and no established screening tools have been specifically designed for this population (28, 29). To address this gap, we previously developed the Child Psychosis-risk Screening System (CPSS), which utilizes the Child Behavior Checklist (CBCL), a comprehensive measure of childhood psycho-behavioral problems (30, 31). The CPSS algorithm was formulated by retrospectively identifying subclinical characteristics during childhood in patients with schizophrenia (32). Specifically, the CPSS risk score is calculated using the T-scores of eight CBCL syndrome subscales, weighted by coefficients derived from a logistic regression model developed in our prior study. A prior cross-sectional study demonstrated that the CPSS has sufficient ability to discriminate schizophrenia spectrum disorders (SSD) from other conditions, including neurodevelopmental disorders (29).

Therefore, this prospective study aimed to validate the ability of the CPSS to predict future SSD status by following a cohort of pediatric and psychiatric outpatients who visited clinics for developmental, psychosomatic, or psychiatric disorders. Further, to confirm the robustness of the CPSS's predictive accuracy and its clinical implementability, we compared the predictive ability of the parsimonious CPSS, which uses only eight CBCL subscales, with that of a complex *post-hoc* machine learning (ML) benchmark model [Light Gradient Boosting Machine (LightGBM)], which maximizes predictive performance. Confirmation of the predictive accuracy of the CPSS would support its implementation in general pediatric practice, thereby making a significant contribution to the early identification and intervention for children at risk of psychosis.

## 2 Materials and methods

### 2.1 Participants

**Figure 1:**
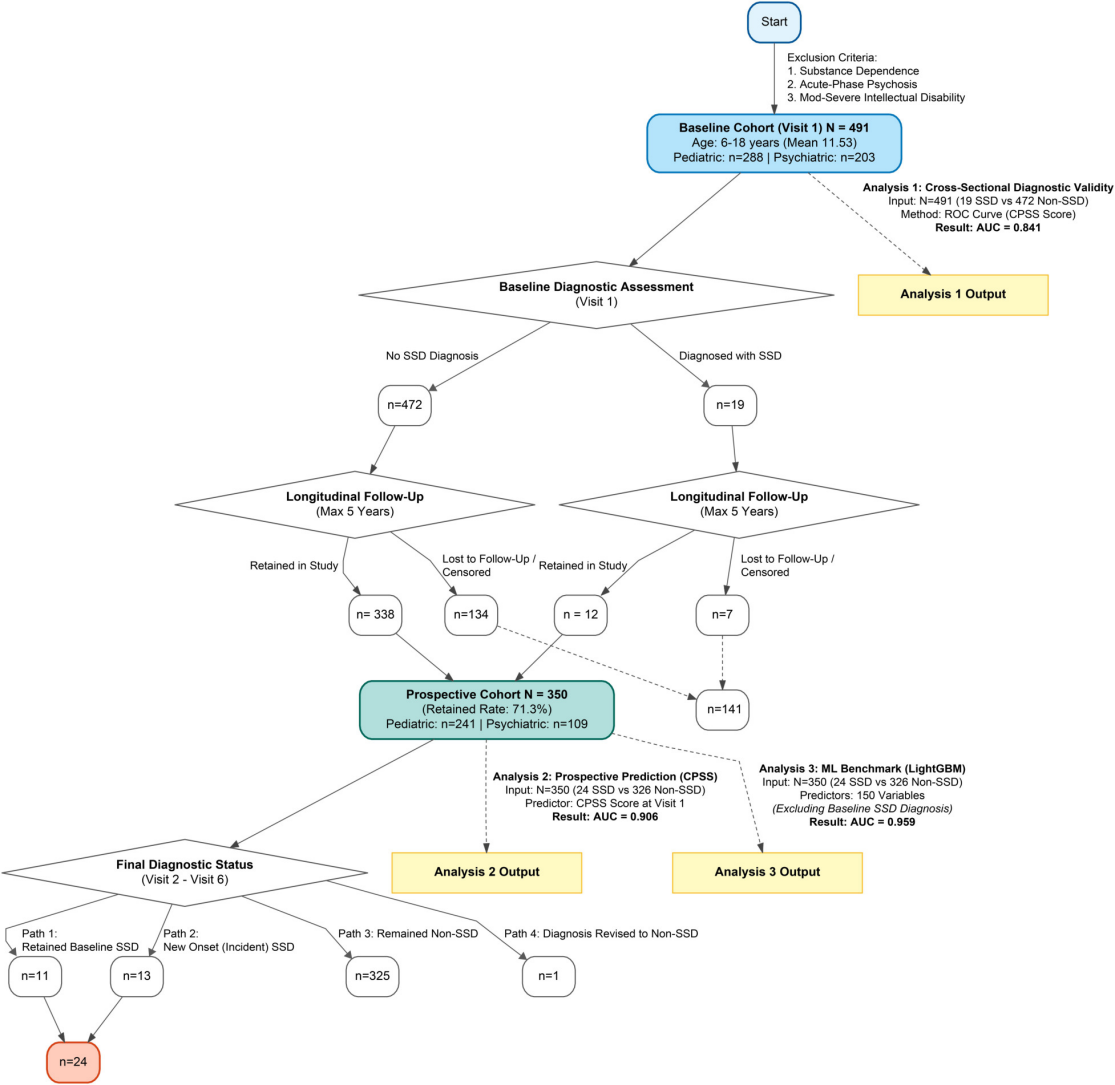
Study flow diagram: the sample size and number of SSD events included in each analysis. SSD, schizophrenia spectrum disorders; CPSS, child psychosis-risk screening system; ROC, receiver operating characteristic; AUC, area under the curve.

The initial cohort (Visit 1) comprised 491 outpatients aged 6–18 years, consecutively recruited from one university and three community hospitals in the Kansai region of Japan between December 2019 and June 2025. This cohort of 491 patients represents the finalized and expanded dataset of a preliminary cohort of 478 patients previously reported at the 33rd European Congress of Psychiatry. The previously reported cohort is included almost entirely in the present analysis, with 478 patients overlapping between the two datasets. This study extends the follow-up period and finalizes the predictive modeling for this cohort. The sample included 288 patients from pediatric developmental clinics and 203 patients from psychiatric clinics. Detailed demographic characteristics are shown in Table 1. Participants underwent annual diagnostic assessments during follow-up for up to 5 years (Visits 2–6). In total, 141 patients were lost to follow-up. Specifically, 85 patients discontinued due to clinical resolution, 36 self-interrupted follow-up, and 20 were administratively censored at the end of the study (Figure 1). For similar reasons, the follow-up duration after Visit 2 varied. From the initial cohort, 350 participants were retained for follow-up, with a mean follow-up duration of 2.26 ± 1.03 years. These 350 patients included 241 patients from pediatric developmental clinics and 109 patients from psychiatric clinics (Supplementary Table S1). There were significantly more non-retained participants from psychiatric clinics than from pediatric developmental clinics, and there was a significant difference in age between retained and non-retained participants.

**Table 1:** Demographic characteristics of patients at visit 1.

Characteristics	All participants ***n*** **(%) or mean**	Pediatric patients ***n*** **(%) or mean**	Psychiatric patients ***n*** **(%) or mean**
Sex (Male/Female)^a^	293/198 (59.6% male)	213/75 (73.9% male)	80/123 (39.4% male)
Age (years)^b^	11.53 ± 3.41	9.57 ± 2.57	14.32 ± 2.35

a Mean ± standard deviation; significant difference between pediatric/psychiatric patients were assessed using Pearson's chi-squared test: *χ*^2^ = 59.06, *p* < 0.001.

b Mean ± standard deviation; significant difference between pediatric/psychiatric patients were assessed using the Student's *t*-test: *t* = 21.12, *p* < 0.001.

In Japan, specialized child and adolescent psychiatric outpatient services are limited; consequently, pediatric developmental clinics often manage a broad range of mental health conditions in children, including psychosomatic disorders, developmental delays, adjustment disorders, and cases of child abuse.

The inclusion criteria were: (i) patients already diagnosed with a developmental disorder, psychosomatic disorder, or psychiatric disorder at study entry; (ii) provision of free and voluntary informed consent (written or verbal); and (iii) eligibility confirmed by the attending research physician. The exclusion criteria were: (i) alcohol or drug use disorder and (ii) acute-phase psychosis or moderate-to-severe intellectual disability, as obtaining informed consent from these patients was considered difficult.

### 2.2 Procedure and measures

At Visit 1, all 491 participants were assessed using the CBCL, and relevant clinical data were collected from their medical records. The onset of SSD was monitored over a maximum follow-up period of 5 years (mean ± standard deviation = 2.26 ± 1.03 years), from the initial assessment (Visit 1) through the fifth year (up to Visit 6). The sample sizes and numbers of participants with SSD at each visit are shown in the study flow diagram (Figure 1).

#### 2.2.1 Diagnostic assessment

Diagnoses were initially made by attending physicians in outpatient clinics and were subsequently confirmed according to the research protocol (Supplementary Table S2). Given the real-world clinical setting of this study, the clinicians and research physicians conducting the diagnostic assessments were not strictly blinded to the patients' baseline CBCL results or CPSS risk scores. All participants were evaluated using the Structured Clinical Interview for DSM-5 Research Version (33) and the Structured Clinical Interview for DSM-IV Childhood Diagnoses (34).

At Visit 1, 19 participants were diagnosed with SSD, including schizophreniform disorder (*n* = 6), brief psychotic disorder (*n* = 2), delusional disorder (*n* = 2), and schizophrenia (*n* = 9). Of these cases, one diagnosis was later revised, and seven participants discontinued participation, resulting in 11 participants with SSD at Visit 1 in the analyzed sample. During the follow-up period from Visit 2 onward, 13 participants received a new diagnosis of SSD, bringing the total to 24 participants with a final diagnosis of SSD (Figure 1). These diagnoses comprised schizophreniform disorder (*n* = 5) and schizophrenia (*n* = 19).

#### 2.2.2 CBCL/6-18

The CBCL is a comprehensive, parent-rated checklist developed by Achenbach et al. to assess emotional, behavioral, and somatic problems in children over the preceding 6 months (30, 31). Based on the raw scores of 120 items, T-scores are calculated for eight syndrome subscales (Anxious/Depressed, Withdrawn/Depressed, Somatic Complaints, Social Problems, Thought Problems, Attention Problems, Rule-Breaking Behavior, and Aggressive Behavior), as well as for Internalizing, Externalizing, and Total Problems scales. Because norms vary by country, sex, and age, subscale scores were converted to T-scores using the appropriate standardized norms. The CBCL was completed at the hospital by one or both parents, yielding one record per child. To prevent missing data, the CBCL was completed using a tablet device that alerted participants when items were incomplete.

#### 2.2.3 Child psychosis-risk screening system

In this study, we used the CPSS, a tool developed in our previous retrospective and cross-sectional studies (29, 32), to identify SSD at Visit 1 and to identify the risk of future SSD status (at follow-up visits). The CPSS calculates a child's psychosis risk score (hereafter referred to as the CPSS risk score) based on the T-scores of the eight CBCL syndrome subscales (Supplementary Appendix S1).

#### 2.2.4 Clinical data

Demographic information (age, sex, and birth month), Diagnostic and Statistical Manual of Mental Disorders, 5th Edition clinical diagnoses, and data on experiences of abuse, bullying, and social withdrawal (*hikikomori*) were collected by the collaborating attending physicians. Birth months were categorized into seasons as follows: spring (March–May), summer (June–August), autumn (September–November), and winter (December–February). Information on pharmacotherapy was categorized as either receiving treatment (*n* = 326) or not receiving treatment (*n* = 165).

Abuse, bullying, and social withdrawal were assessed using *ad hoc* scales. Abuse was classified dichotomously as present (*n* = 69) or absent (*n* = 422). Bullying victimization was rated on a 4-point scale (0: not at all true, 1: sometimes true, 2: often true, 3: always true). Social withdrawal (*hikikomori*) was also rated on a 4-point scale (0: not at all true [rarely absent from school], (1): sometimes true [occasionally absent from school], (2): often true [absent from school for over half a month and rarely goes out], (3): always true [not attending school for over 6 months and rarely leaves home]). To prevent missing data, all clinical variables were completed using a tablet device that alerted participants when items were incomplete.

### 2.3 Statistical analysis

To evaluate the ability of the CPSS risk score to predict an SSD diagnosis, receiver operating characteristic (ROC) curve analyses were conducted, with SSD status at Visit 1 and at Visit 2 or later as the outcomes. The areas under the curve (AUCs) were calculated using the non-parametric trapezoidal method. The cutoff value was defined as the point on the ROC curve at which sensitivity and specificity were optimized. We first examined the immediate diagnostic ability of the CPSS for SSD at Visit 1 (*n* = 491), followed by its prognostic ability for SSD diagnosis during the follow-up period (final diagnosis after Visit 2, *n* = 350).

Next, we developed a *post-hoc* ML model to predict SSD status during the follow-up period. This model was constructed as a benchmark to assess the parsimony and effectiveness of the CPSS. We selected LightGBM (35), a gradient-boosted decision tree algorithm, as the primary ML model because it has demonstrated strong performance and robustness for tabular data with non-linear relationships, while also enabling model interpretability using SHapley Additive exPlanations (SHAP). This model included a total of 150 variables: the CPSS risk score at Visit 1; raw scores for all CBCL items (Q1–113); all CBCL T-scores (Total, Internalizing, Externalizing, and the eight syndrome scales); the difference between Internalizing and Externalizing T-scores; and all aforementioned clinical data (Supplementary Table S3). The target outcome included all 24 participants with a final SSD diagnosis during the study period, including the 11 prevalent cases diagnosed at Visit 1 and the 13 incident cases diagnosed during follow-up from Visit 2 onward. To prevent data leakage and ensure that the model did not trivially rely on pre-existing diagnostic labels, SSD diagnosis at Visit 1 was explicitly excluded as a predictor. This exclusion ensured that the prediction task focused on identifying individuals who would develop SSD after Visit 2 using only CBCL data and clinical data other than SSD diagnostic status.

Furthermore, given the low event rate (class imbalance) of approximately 6.8% (24/350), which can inflate traditional AUC estimates due to the dominance of true negatives, we evaluated model performance using precision–recall curves (Supplementary Figure S1) and ensured robust internal validity by utilizing a nested stratified cross-validation scheme that strictly preserved the proportion of SSD cases across all folds. This optimized model performance while avoiding overfitting and estimated the out-of-sample predictive performance. Model performance was evaluated using an outer five-fold stratified cross-validation repeated five times, preserving the proportion of SSD and non-SSD cases in each fold. Hyperparameters for all ML models were tuned using an inner three-fold stratified cross-validation to maximize the mean AUC. For interpretability analyses, including SHAP values and partial dependence plots, a final LightGBM model was refitted using the full dataset.

For benchmarking purposes, we additionally trained Random Forest and Decision Tree classifiers (36, 37) using the same set of predictors and the same stratified k-fold cross-validation procedure as used for the LightGBM model.

To evaluate variable importance for SSD prediction in the *post-hoc* LightGBM model, SHAP values (38, 39) were compared across features. SHAP values calculate the directional contribution of each feature to the final prediction; a positive SHAP value increases the predicted probability of the outcome, while a negative value decreases it. Furthermore, partial dependence plot (PDP) analysis (40) was conducted to examine the average marginal effects of the CPSS risk score and other variables on the predicted probability of SSD.

All statistical analyses were performed using SPSS for Windows (version 30.0; IBM Corp., Armonk, NY, USA). ML analyses—including LightGBM, Random Forest, and Decision Tree models, as well as SHAP and partial dependence plots—were implemented in Python using the lightgbm, scikit-learn, and shap libraries. Regarding CBCL items and all other clinical variables, only complete case data were used for all analyses, so data with missing values were not included. The significance level was set at *p* < 0.05 for all tests.

### 2.4 Ethics statement

This study was reviewed and approved by the Ethics Committee of Kyoto Women's University and Shiga University of Medical Science, Japan. Written informed consent to participate in this study was obtained from the participants' legal guardians or next of kin.

## 3 Results

### 3.1 Discriminative and predictive ability of the CPSS risk score: cross-sectional and prospective ROC analyses

**Figure 2:**
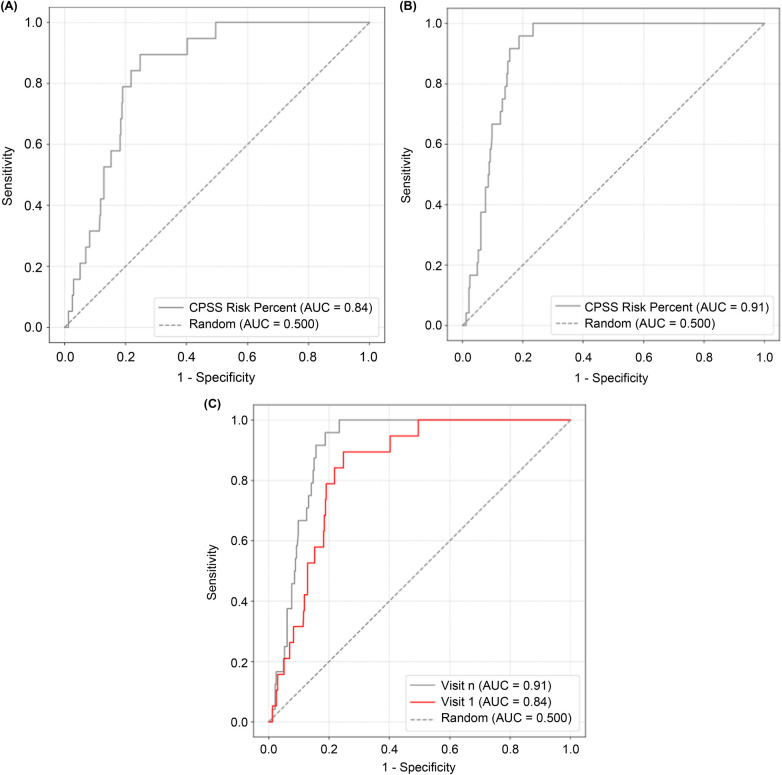
Performance of the CPSS risk score in identifying current SSD and predicting future SSD. **(A)** Receiver operating characteristic curve showing the concurrent discriminative performance of the CPSS risk score for SSD at baseline (Visit 1; *n* = 491). **(B)** ROC curve illustrating the predictive performance of the baseline CPSS risk score for subsequent SSD status during follow-up (final diagnosis from Visit 2 onward; *n* = 350). **(C)** Comparison of ROC curves demonstrating better performance for prediction than for identifying current SSD. Visit 1, ROC curve of the CPSS algorithm for the concurrent SSD status; Visit n, ROC curve of the CPSS algorithm for future SSD status (Visit 2–6). CPSS, child psychosis-risk screening system; SSD, schizophrenia spectrum disorders; ROC, receiver operating characteristic; AUC, area under the curve.

First, the ability of the CPSS risk score to discriminate SSD cases at baseline (Visit 1) was evaluated using ROC analysis (*n* = 491). The CPSS demonstrated sufficient discriminative performance [AUC = 0.841, 95% confidence interval (CI): 0.781–0.900], with a cutoff of 95.65% yielding a sensitivity of 89.5% and a specificity of 75.2% (Figure 2A). Next, we assessed the ability of the baseline CPSS risk score to predict the subsequent SSD status during the follow-up period (final diagnosis from Visit 2 onward; *n* = 350). The CPSS risk score showed excellent predictive performance (AUC = 0.906, 95% CI: 0.87–0.94), with an optimal cutoff of 98.14% providing a sensitivity of 91.7% and a specificity of 84.4% (Figure 2B). Notably, the predictive ability of the CPSS risk score for future SSD status exceeded its concurrent discriminative ability (Figure 2C). Significant patient attrition from baseline to follow-up may have introduced bias, potentially affecting the prognostic AUC estimate.

### 3.2 Comparison of predictive performance: CPSS-only model vs. *post hoc* machine learning model

**Figure 3:**
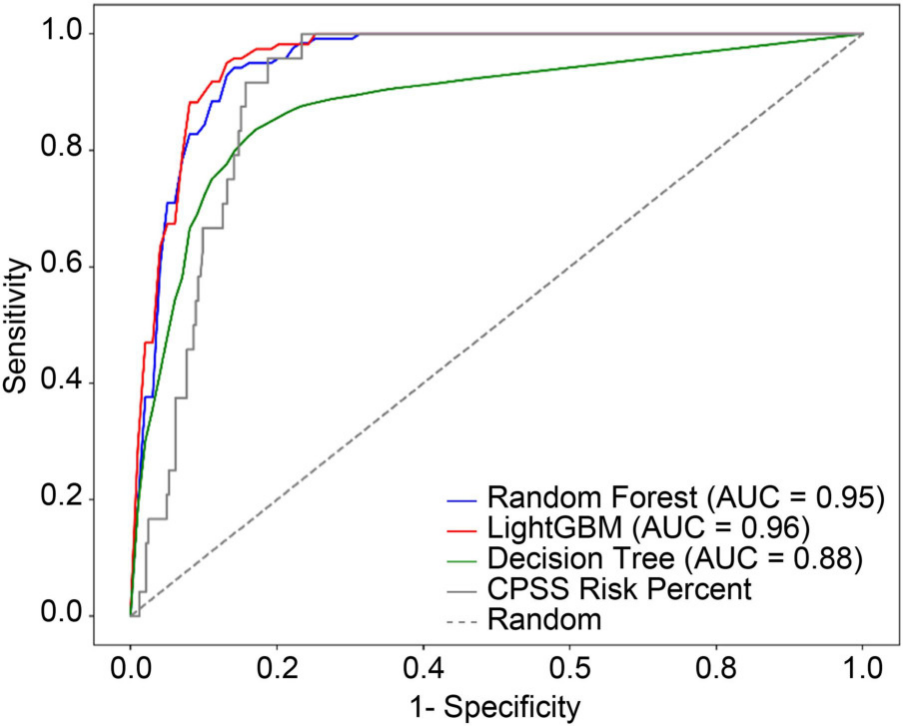
Comparison of predictive performance across models. ROC curves for the CPSS, LightGBM model, Random Forest model, and single Decision Tree model in predicting SSD status during follow-up (final diagnosis from Visit 2 onward; *n* = 350 in all). ROC, receiver operating characteristic; CPSS, child psychosis-risk screening system; LightGBM, light gradient boosting machine; SSD, schizophrenia spectrum disorders; AUC, area under the curve.

The *post hoc* ML (LightGBM) model (*n* = 350), which incorporated all 150 baseline variables (e.g., CPSS risk score and clinical data), demonstrated strong predictive performance for subsequent SSD status in repeated stratified k-fold cross-validation, with an AUC of 0.959 (95% CI: 0.95–0.97). At the optimal cutoff for the predicted probability of SSD, the model achieved a sensitivity of 95.0% and a specificity of 86.9% (Figure 3). For comparison, conventional tree-based models (Random Forest and a single Decision Tree) trained using the same predictors did not outperform the LightGBM model in terms of AUC (Figure 3).

Although the performance of LightGBM was slightly higher than that of the CPSS-only model (AUC = 0.906), the difference in AUC between the two models was modest. However, at their respective optimal cutoffs, the CPSS-only model showed lower precision, reflecting a higher number of false-positive classifications compared with the LightGBM model (Supplementary Figure S1).

### 3.3 Feature importance in the ML model

**Figure 4:**
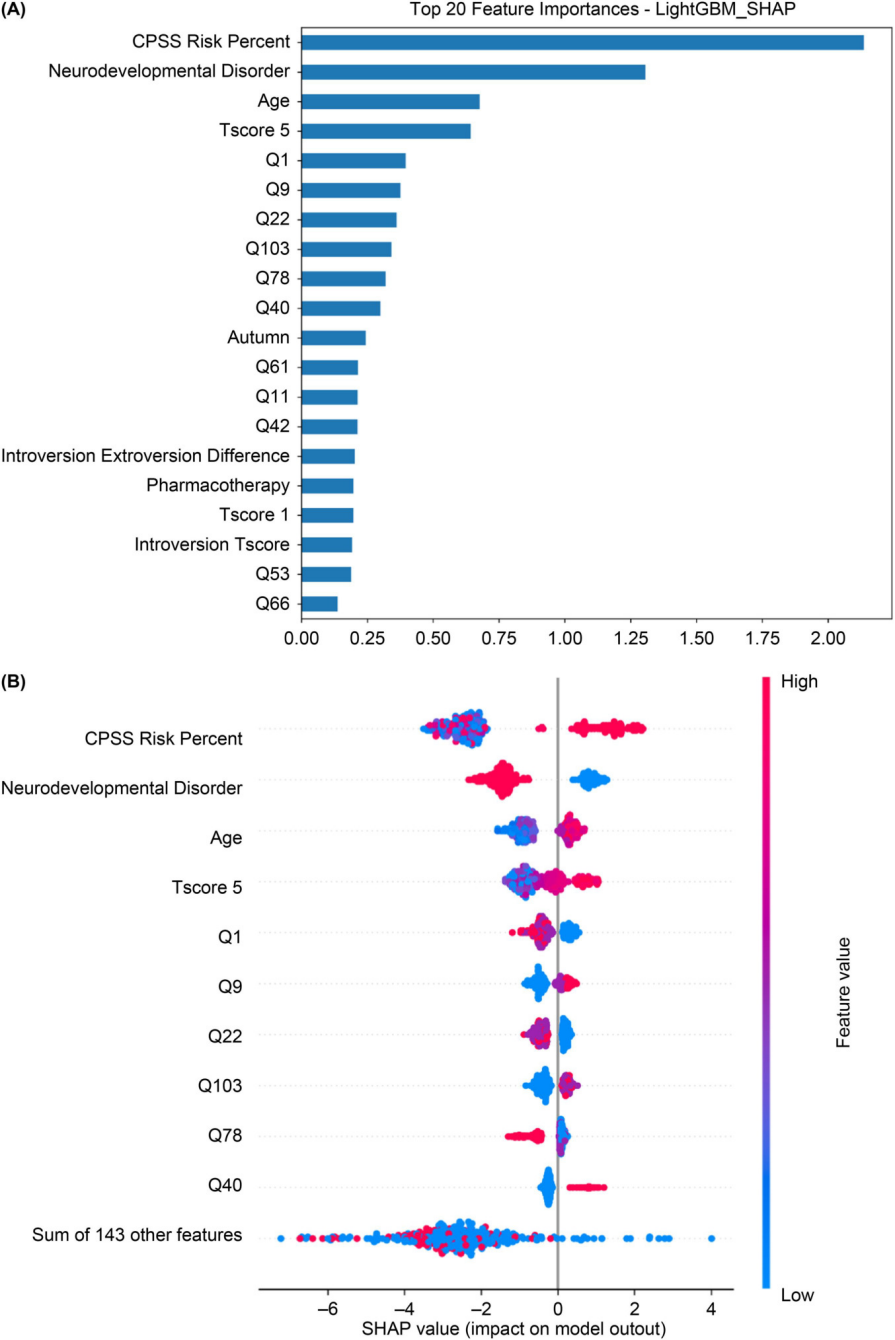
SHAP-based feature importance analysis in the LightGBM model. **(A)** Feature importance ranked by mean absolute SHAP values across predictors. **(B)** SHAP summary plot illustrating the impact of top predictive features on the predicted probability of SSD status. Each point represents an individual participant; color indicates feature value (red: high, blue: low), and horizontal position (SHAP value) indicates contribution to prediction. (“Q” labels refer to individual CBCL item scores, and “Tscore” labels refer to syndrome subscales. The model incorporated 150 baseline clinical variables, which expanded to 153 features after one-hot encoding categorical variables, resolving the sum of 10 individual features and 143 grouped features.) SHAP, shapley additive explanations; LightGBM, light gradient boosting machine; SSD, schizophrenia spectrum disorders.

We evaluated the contribution of each variable to the model's predictions using SHAP values, with feature importance calculated as the mean absolute SHAP value. The analysis revealed that the CPSS risk score was the most influential feature for predicting SSD status. In contrast, the CBCL “Thought Problems” (T-score 5), traditionally considered a key indicator of psychosis risk in children, showed lower feature importance (Figures 4A,B).

### 3.4 Partial dependence plot analysis of key predictors

The effects of key variables on the model's predictions were visualized using PDPs. The analysis suggested the presence of threshold effects, with the predicted probability of SSD increasing sharply and non-linearly when the CPSS risk score exceeded 94.83% (Figure 5). In contrast, other variables—such as age and CBCL T-scores, including “Thought Problems” (T-score 5) —exhibited more linear relationships with the predicted probability of SSD (Figure 6, Supplementary Figures S2–S12).

**Figure 5:**
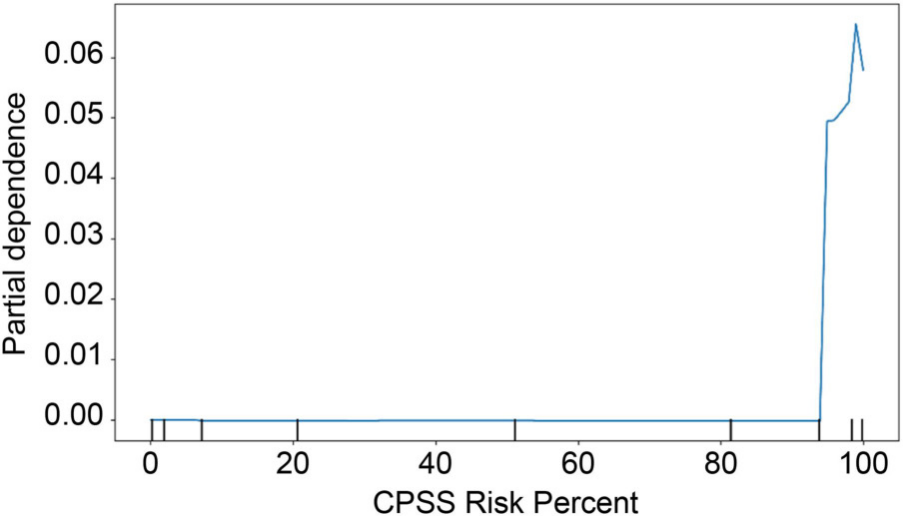
Partial dependence plot showing the relationship between the CPSS risk score and the predicted probability of SSD status in the LightGBM model. CPSS, child psychosis-risk screening system; SSD, schizophrenia spectrum disorders.

**Figure 6:**
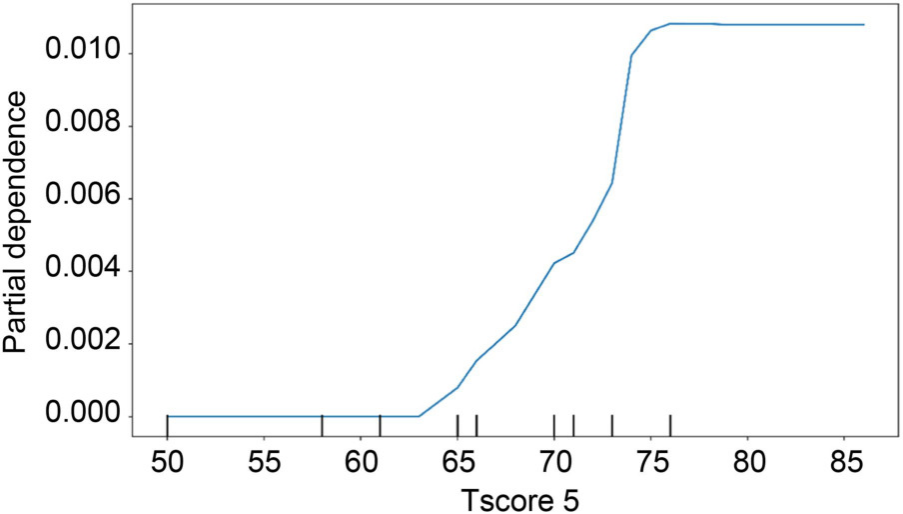
Partial dependence plot showing the relationship between the CBCL “Thought Problems” T-score (T-score 5) and the predicted probability of schizophrenia Spectrum disorder (SSD) status in the LightGBM model. CBCL, child behavior checklist; SSD, schizophrenia spectrum disorders; LightGBM, light gradient boosting machine.

## 4 Discussion

The primary objective of this prospective study was to validate the predictive ability of the CPSS for future SSD status. Our findings demonstrate that the CPSS risk score alone showed good predictive performance for the final SSD status (AUC = 0.906). These results suggest that the CPSS may function as a practical and effective tool for objectively identifying children with high risk who warrant early psychiatric referrals, particularly in non-specialized clinical settings, such as general pediatrics. Regarding the external validity of our findings in primary care settings, it is essential to consider the structural context of the healthcare system. In many countries, including Japan, there is a critical shortage of specialized child and adolescent psychiatric services. As a result, general pediatricians and pediatric developmental clinics act as the de facto primary care gatekeepers for pediatric mental healthcare. Children in the prodromal phase of SSD rarely present with overt psychosis symptoms; instead, they typically present to pediatric primary care settings with non-specific behavioral changes, school refusal, or medically unexplained somatic complaints. Because our study cohort heavily consisted of patients from exactly these pediatric developmental clinics, which act as the first point of clinical contact, our findings strongly support the external validity and practical utility of the CPSS as a primary care screening tool. Implementing the CPSS in such front-line pediatric settings can provide a standardized metric to identify latent psychosis risk, thereby optimizing the limited psychiatric resources using evidence-based, streamlined referral pathways.

Notably, the performance of the CPSS demonstrated that its predictive validity (AUC = 0.906) exceeded its concurrent diagnostic validity (AUC = 0.841). This finding challenges the traditional reliance on cross-sectional criteria for early detection. Typically, diagnostic tools are designed to capture pathology present at the time of assessment, resulting in higher cross-sectional diagnostic concordance. In contrast, the CPSS showed a divergence in which predictive accuracy surpassed concurrent accuracy. This phenomenon likely reflects the pathophysiological characteristics of the prodromal phase of SSD (41). At the time of evaluation, children at risk may not fully meet the diagnostic criteria (e.g., Diagnostic and Statistical Manual of Mental Disorders, 5th Edition) for SSD and instead present with subthreshold symptoms. Consequently, a cross-sectional “immediate diagnosis” by a clinician may result in a non-SSD classification, creating a discrepancy with the CPSS risk classification and contributing to a relatively lower AUC for concurrent diagnosis. In contrast, the CPSS, which integrates combinations of CBCL scales, may capture subtle behavioral changes and vulnerabilities during the prodromal stage more sensitively than the overt psychotic symptoms required for a definitive diagnosis. Given that psychosis onset is often gradual and accompanied by diagnostic instability (42), the CPSS appears particularly well suited to capture the dynamic changes that precede onset. In other words, the CPSS is structured to be more sensitive to forecasting future onset than to identifying current diagnosis, with its predictive accuracy substantiated over time. An AUC of 0.906 is highly favorable for a screening instrument and supports its potential clinical utility.

In this study, we also retrospectively constructed a more complex ML prediction model using LightGBM. Although the model incorporating all 150 variables showed superior predictive performance than the CPSS (AUC = 0.959), the difference was limited. This finding implies that the CPSS risk score—derived from combinations of CBCL scales—captures substantially more critical information than numerous other clinical variables and is sufficient for accurate prediction, even when compared with a high-dimensional, non-linear model. In this respect, the scientific merit of the CPSS's parsimony is particularly evident.

Consistent with this interpretation, the variable-importance analysis showed that the CPSS risk score contributed markedly more than any variable. In contrast, the CBCL “Thought Problems” subscale, which has traditionally been emphasized as an indicator of psychotic risk in children, exhibited relatively lower importance. This is a notable finding that prompts reconsideration of conventional risk assessment approaches. Historically, the CBCL “Thought Problems” subscale has been regarded as a strong marker of psychosis risk because it reflects auditory hallucinations and unusual thoughts (43–45). However, in our analysis, the CPSS risk score demonstrated greater predictive importance than this single subscale.

These findings highlight the limitations of focusing exclusively on single specific symptoms (e.g., hallucinations or delusion-like experiences) when predicting SSD. Rather, SSD risk appears to manifest as a constellation of dysfunctions across multiple domains—not only thought disturbances but also emotional dysregulation, social withdrawal, and somatic anomalies (46). The CPSS effectively captures this multidimensional pattern through an integrated algorithm, thereby exerting stronger predictive power than any individual scale. This mathematically supports the importance of conceptualizing psychopathology not as a mere “list of symptoms” but as a holistic pattern (29).

Furthermore, PDP analysis revealed a non-linear relationship in which the predicted probability of SSD rose sharply once the CPSS risk score exceeded a specific threshold (approximately 95%). This finding suggests that risk does not increase merely in a linear fashion; rather, when pathology crosses a critical “tipping point”, the likelihood of onset escalates markedly (47). This threshold may therefore serve as an objective criterion for defining high-risk groups in clinical practice.

This study offers a concrete action framework for pediatric and primary care settings. For children who repeatedly present with medically unexplained physical symptoms (psychosomatic complaints) (48), routine administration of the CBCL and evaluation via the CPSS allows the need for psychiatric referral to be expressed as an objective probability. This approach may reduce the stigma associated with psychiatric consultation for both children and their guardians, thereby facilitating evidence-based referral. Even when an immediate diagnosis is not warranted, a high CPSS risk score indicates a high probability of an “at-risk mental state” (49–51) rather than an absence of abnormality. Communicating this perspective enables closer monitoring and preventive interventions (e.g., stress management, environmental adjustment), rather than passive observation. Moreover, the CPSS can serve as a “shared language” between pediatrics and psychiatry. When referral letters from pediatricians indicate a high CPSS risk score, receiving psychiatrists can more readily suspect a prodromal phase of SSD at the initial visit, thereby expediting consideration of early intervention services. This process improvement is directly linked to shortening the DUP, a key determinant of prognosis (52).

While the CPSS exhibits high predictive accuracy, the clinical implementation of this screening tool requires careful consideration of how high-risk status is communicated to families. Disclosing a “high risk for psychosis” status can have profound psychological effects, potentially inducing internalized stigma and hopelessness in adolescents during a critical period of identity formation. For parents, such disclosure may trigger associative stigma, guilt, and excessive anxiety, which can elevate family tension and inadvertently worsen the child's clinical state. To mitigate these risks, primary care clinicians must adopt destigmatizing communication strategies. Recent studies in Japan have highlighted the effectiveness of culturally sensitive terminology; for instance, framing the condition as “ARMS-kokoro” (At-Risk Mental State of the Mind) rather than explicitly using the term “psychosis” significantly improved family acceptance and reduced stigma (53). Clinicians utilizing the CPSS should avoid deterministic predictions. Instead, high CPSS scores should be communicated through a stress-vulnerability framework, explaining that the child is currently experiencing a heightened sensitivity to stress, and the focus should remain on managing present distress (e.g., somatic complaints, anxiety) and providing family psychoeducation to foster a supportive, low-stress home environment.

Moreover, there are promising prospects for precision psychiatry through the application of ML. Our *post-hoc* LightGBM model estimates risk by considering not only basic attributes such as sex and age but also individual environmental factors, including bullying and maltreatment. In other words, it evaluates risk within the individual context of each child rather than against a population average. Consequently, the LightGBM model demonstrated higher predictive ability and precision for SSD onset than those of the CPSS. With further development, the CPSS algorithm may evolve to incorporate these individual-level factors, becoming a more sophisticated system capable of proposing intervention strategies tailored to specific risk profiles (e.g., prioritizing anti-bullying interventions or considering pharmacotherapy). Furthermore, future iterations of the CPSS could directly incorporate established environmental risk factors—such as childhood abuse, neglect, peer victimization, and winter birth—to further refine risk stratification and improve predictive performance.

There are several limitations to this study. First, there were analytical limitations. Significant participant loss to follow-up (491 enrolled vs. 350 retained) may have introduced bias. Specifically, the substantially higher attrition among participants from psychiatric clinics compared to pediatric clinics may have systematically shifted the retained cohort towards a milder case mix. This imbalance could alter the underlying SSD prevalence, potentially affecting the validity of the AUC estimates and the generalizability of our findings. Similarly, the predictive AUC estimates were based on binary outcome classifications under variable follow-up periods and were therefore potentially subject to censoring-related bias, meaning that the superiority of the predictive AUCs over that of the concurrent AUCs may have been affected by the follow-up structure. Furthermore, we must note a methodological limitation regarding outcome assessment. The clinicians and research physicians who confirmed the SSD diagnoses using the Structured Clinical Interview for DSM protocols were not completely blinded to the baseline CBCL and CPSS results. As per the TRIPOD and STARD guidelines, lack of blinding can introduce confirmation bias, potentially inflating the predictive accuracy if clinicians unconsciously lower the threshold for diagnosis in patients with high screening scores. However, we believe that this bias was minimized because the predictor variables (CBCL) were strictly parent-rated and objective to the clinician, and the outcome diagnoses relied on highly structured, criteria-driven Structured Clinical Interview for DSM interviews rather than unstructured clinical impressions. Second, the CPSS demonstrated lower precision than the ML model, resulting in a higher rate of false positives. While it remains possible that some of these false-positive cases may become true positives with longer-term follow-up, the potential risks of unnecessary labeling and excessive medical intervention must be considered at this stage. On the other hand, as previously noted, the CPSS risk score may capture subtle changes and vulnerabilities in the prodromal stage more sensitively than it captures overt psychiatric symptoms. It should be noted that individuals identified as high risk by exceeding the CPSS cutoff value likely possess an underlying vulnerability, such that mental health problems may emerge when exposed to stressors such as academic or interpersonal demands. Accordingly, the CPSS should be used not as a diagnostic tool but strictly as a screening instrument for identifying risk groups. Third, the follow-up period in this study was limited to a maximum of 5 years, which may be insufficient to capture SSD onset occurring in late adolescence or early adulthood. Longer-term follow-up studies are therefore required, including continued monitoring of false-positive cases. Finally, this study was conducted in a single region in Japan (the Kansai region), and further validation is needed to confirm the effectiveness and generalizability of the CPSS across different cultures and healthcare systems.

In conclusion, the CPSS is a simple, practical, and clinically useful screening tool for predicting the onset of SSD in children. The key findings can be summarized as follows: (1) the predictive ability of the CPSS exceeded its concurrent diagnostic capability, indicating that it sensitively captures not only manifest symptoms but also latent vulnerabilities and prodromal features associated with future onset; and (2) ML analyses confirmed that the comprehensive risk assessment provided by the CPSS was a more powerful predictor than conventional approaches relying solely on psychiatric symptoms, such as the CBCL “Thought Problems” scale. Collectively, these findings strongly support the CPSS as an objective and reliable auxiliary screening instrument for the early identification of the SSD risk in children presenting with psychosomatic symptoms in primary care settings. Implementation of the CPSS may play a pivotal role in connecting overlooked children with a high risk of SSD to specialized psychiatric care at an early stage, thereby facilitating early intervention aimed at preventing disease onset and improving prognosis.

## Data Availability

The raw data supporting the conclusions of this article will be made available by the authors, without undue reservation.
