# Supplementary material for: Predicting schizophrenia spectrum disorders in pediatric outpatients: a prospective validation of the child psychosis-risk screening system
Source: Front Child Adolesc Psychiatry. 2026 Jun 17;5:1840330. doi: 10.3389/frcha.2026.1840330 (PMC13319075; doi:10.3389/frcha.2026.1840330)
Supplement: Supplementary file 1 [file Table1.docx]

Supplementary Material

## 1 Supplementary Figures

**Supplementary Figure 1.** PR curve of the Child Psychosis-risk Screening System (CPSS), tree-based models (Random Forest and a single Decision Tree), and the Light Gradient Boosting Machine (LightGBM) model. PR curve, precision–recall curve


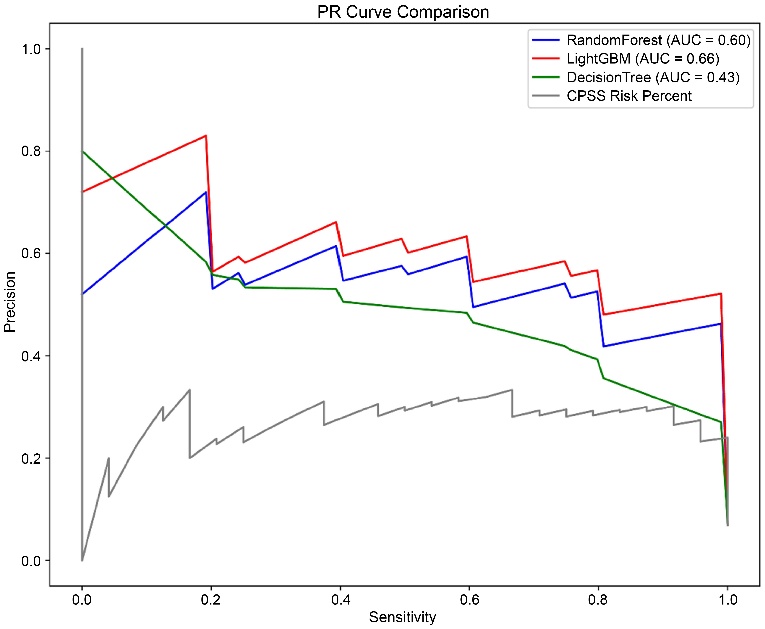


**Supplementary Figure 2.** Partial dependence plot showing the relationship between the Child Behavior Checklist (CBCL) “Anxious/Depressed” T-score (T-score 1) and the predicted probability of onset of schizophrenia spectrum disorders (SSD) in the Light Gradient Boosting Machine (LightGBM) model


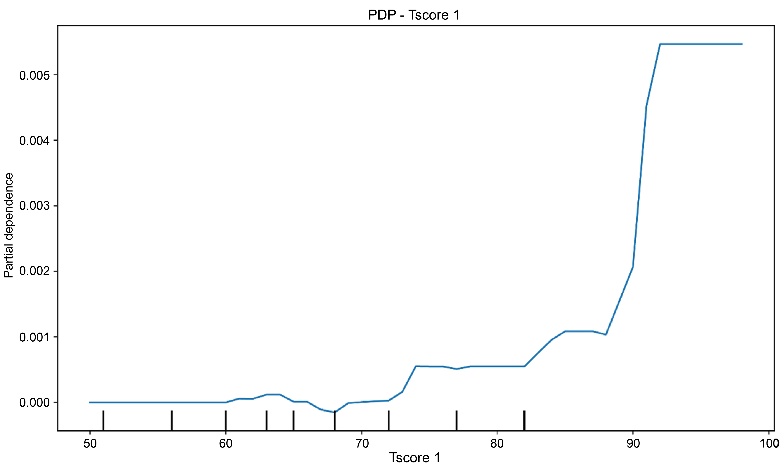


**Supplementary Figure 3.** Partial dependence plot showing the relationship between the Child Behavior Checklist (CBCL) “Withdrawn/Depressed” T-score (T-score 2) and the predicted probability of onset of schizophrenia spectrum disorders (SSD) in the Light Gradient Boosting Machine (LightGBM) model


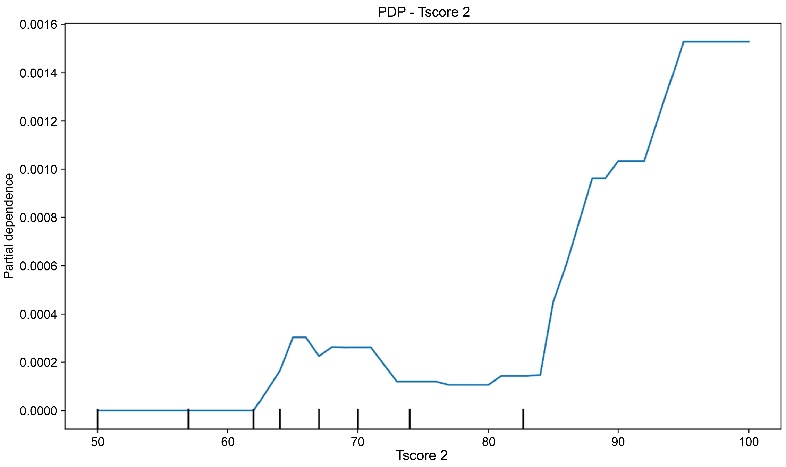


**Supplementary Figure 4.** Partial dependence plot showing the relationship between the Child Behavior Checklist (CBCL) “Somatic Complaints” T-score (T-score 3) and the predicted probability of onset of schizophrenia spectrum disorders (SSD) in the Light Gradient Boosting Machine (LightGBM) model


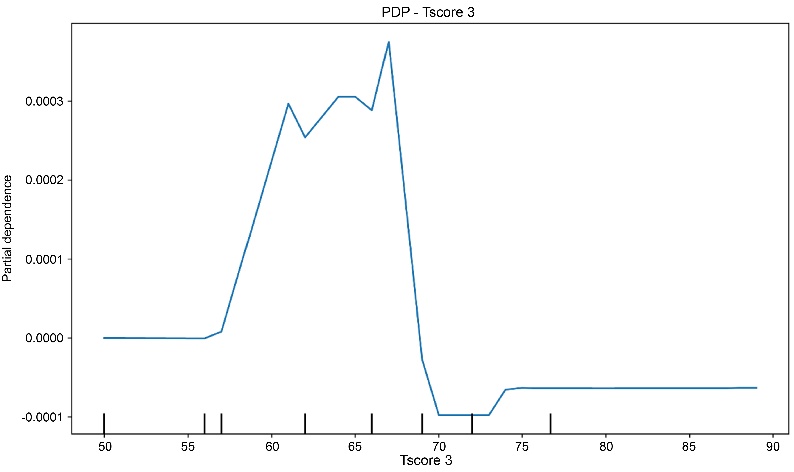


**Supplementary Figure 5.** Partial dependence plot showing the relationship between the Child Behavior Checklist (CBCL) “Social Problems” T-score (T-score 4) and the predicted probability of onset of schizophrenia spectrum disorders (SSD) in the Light Gradient Boosting Machine (LightGBM) model


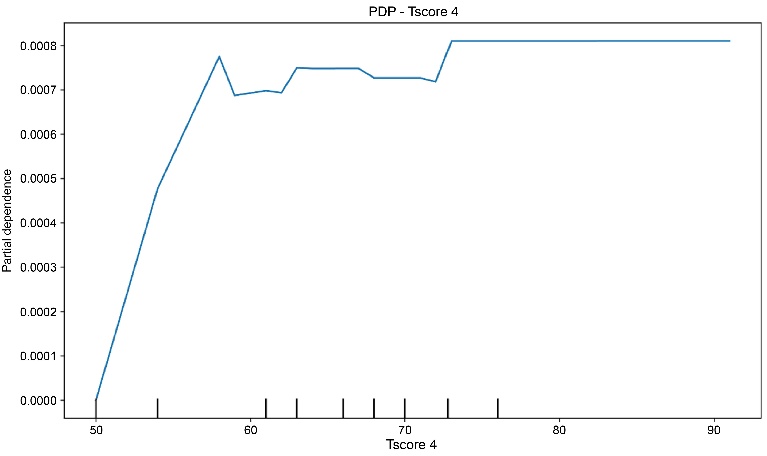


**Supplementary Figure 6.** Partial dependence plot showing the relationship between the Child Behavior Checklist (CBCL) “Attention Problems” T-score (T-score 6) and the predicted probability of onset of schizophrenia spectrum disorders (SSD) in the Light Gradient Boosting Machine (LightGBM) model


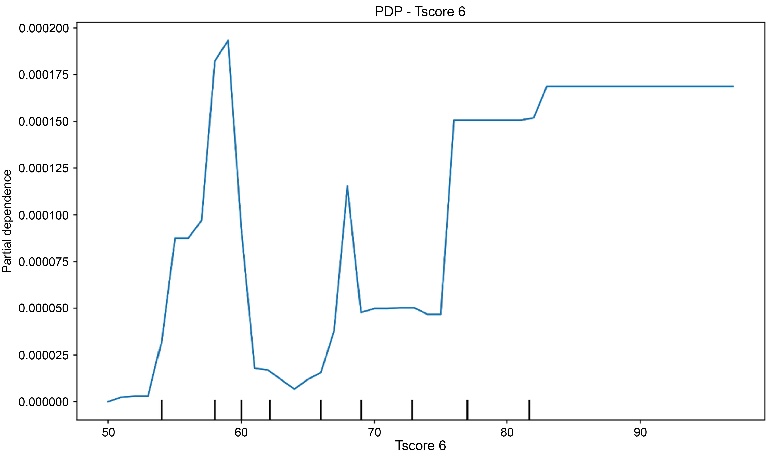


**Supplementary Figure 7.** Partial dependence plot showing the relationship between the Child Behavior Checklist (CBCL) “Rule-Breaking Behavior” T-score (T-score 7) and the predicted probability of onset of schizophrenia spectrum disorders (SSD) in the Light Gradient Boosting Machine (LightGBM) model


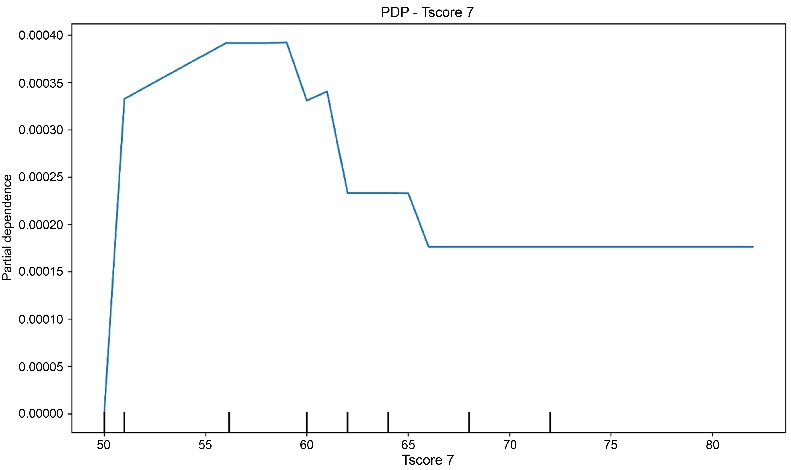


**Supplementary Figure 8.** Partial dependence plot showing the relationship between the Child Behavior Checklist (CBCL) “Aggressive Behavior” T-score (T-score 8) and the predicted probability of onset of schizophrenia spectrum disorders (SSD) in the Light Gradient Boosting Machine (LightGBM) model


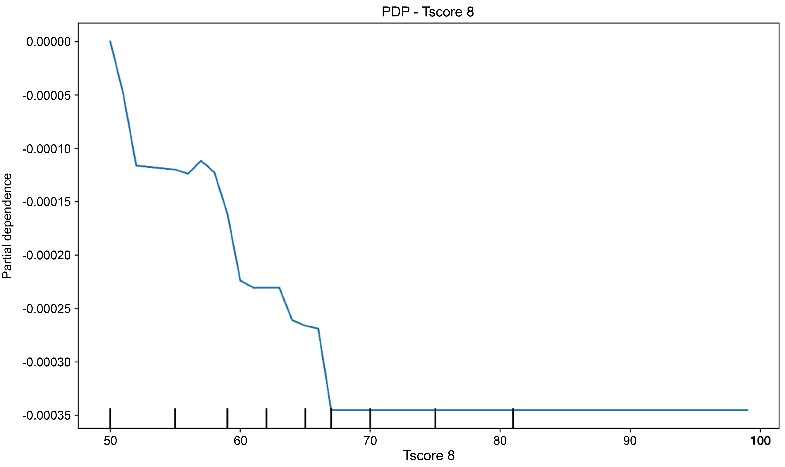


**Supplementary Figure 9.** Partial dependence plot showing the relationship between the Child Behavior Checklist (CBCL) “Internalizing Problems” T-score and the predicted probability of onset of schizophrenia spectrum disorders (SSD) in the Light Gradient Boosting Machine (LightGBM) model


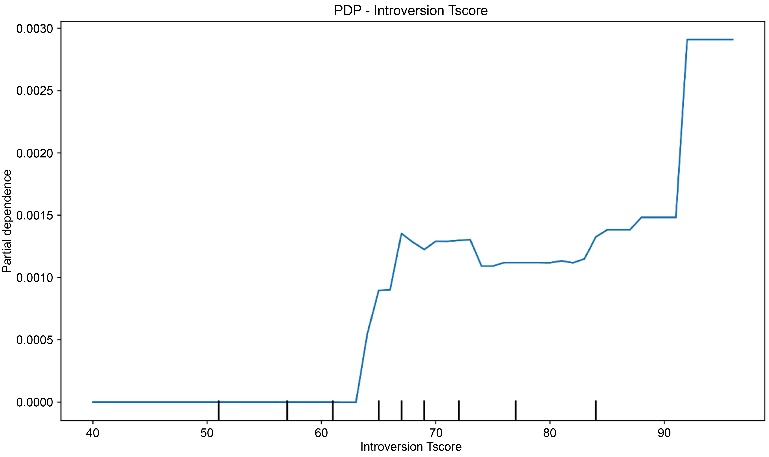


**Supplementary Figure 10.** Partial dependence plot showing the relationship between the Child Behavior Checklist (CBCL) “Externalizing Problems” T-score and the predicted probability of onset of schizophrenia spectrum disorders (SSD) in the Light Gradient Boosting Machine (LightGBM) model


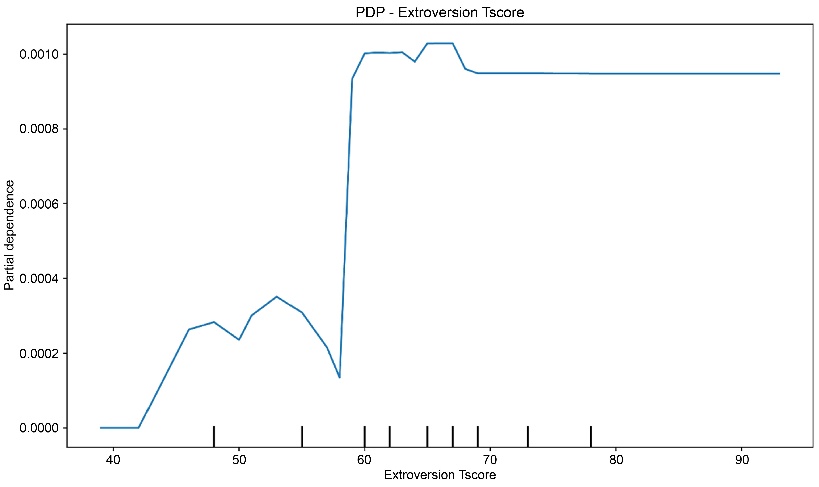


**Supplementary Figure 11.** Partial dependence plot showing the relationship between the Child Behavior Checklist (CBCL) “Total Problems” T-score and the predicted probability of onset of schizophrenia spectrum disorders (SSD) in the Light Gradient Boosting Machine (LightGBM) model


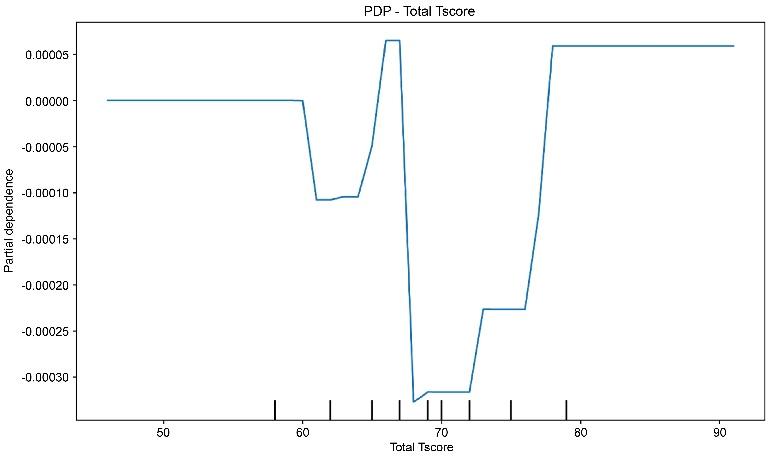


**Supplementary Figure 12.** Partial dependence plot showing the relationship between the Child Behavior Checklist (CBCL) “Age” and the predicted probability of onset of schizophrenia spectrum disorders (SSD) in the Light Gradient Boosting Machine (LightGBM) model


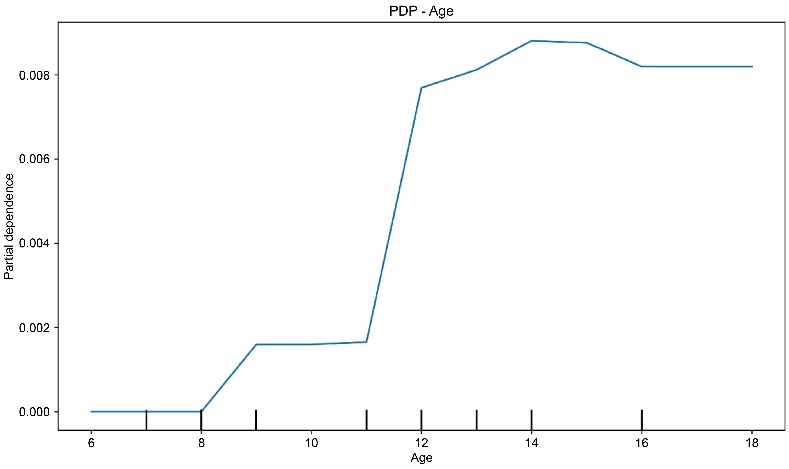


## 2 Supplementary Tables

**Supplementary Table 1. Demographic characteristics of retained/non-retained patients at Visit 1**

|  | Retained participants n=350 | Non-retained participants n=141 |
| --- | --- | --- |
| Sex (Male/Female)^a^ | 217/133  62.0% male | 76/65  53.9% male |
| Age (in years)^b^ | 10.96±3.37 | 12.96±3.07 |
| Pediatric/Psychiatric patients^c^ | 241 (177 male)/109 (40 male) | 47 (36 male)/94 (40 male) |
| Diagnosis of schizophrenia spectrum disorder^d^ | 12  3.42% with diagnosis | 7  4.96% with diagnosis |

^a^ No significant difference between retained/non-retained patients. Pearson’s chi-squared test: χ^2^ = 2.74, p = 0.098. ^b^ Significant difference between retained/non-retained patients. Student's t-test: t = 6.36, p < 0.001. ^c^ Significant difference between retained/non-retained patients. Pearson’s chi-squared test: χ^2^ = 53.30, p < 0.001. ^d^ No significant difference between retained/non-retained patients. Pearson’s chi-squared test: χ^2^ = 0.637, p = 0.425

**Supplementary Table 2. Diagnoses at Visit 1**

|  | **Pediatric patients**  n=288 | **Psychiatric patients**  n=203 |
| --- | --- | --- |
| Sex (Male/Female)^a^ | 213/75  73.9% male | 80/123  39.4% male |
| Age (in years)^b^ | 9.57±2.57 | 14.32±2.35 |
| **Diagnosis ^c^** | **n** | **n** |
| Neurodevelopmental disorders | 260 | 67 |
| Schizophrenia spectrum disorders^c^ | 0 | 19 |
| Bipolar disorders | 0 | 3 |
| Depressive disorders | 8 | 115 |
| Anxiety disorders | 14 | 10 |
| Obsessive-compulsive disorders | 9 | 10 |
| Posttraumatic stress disorder | 3 | 0 |
| Somatic symptom disorders | 0 | 16 |
| Physical disorders (including psychosomatic disorders) | 58 | 0 |
| Epilepsy | 6 | 3 |
| Others | 37 | 19 |

^a^ Significant difference between pediatric/psychiatric patients. Pearson’s chi-squared test: χ2 = 59.06, p < 0.001. ^b^ Mean ± standard deviation; significant difference between pediatric/psychiatric patients. Student's t-test: t = 21.12, p < 0.001. ^c^ Duplicate diagnoses present. ^c^ Schizophreniform disorder (n=6), brief psychotic disorder (n=2), delusional disorder (n=2), schizophrenia (n=9)

**Supplementary Table 3. The 150 variables incorporated into the machine learning models**

| Feature Name | Description |
| --- | --- |
| Sex | Male/female |
| Age | Age at the time of answering the CBCL checklist |
| Q1–Q113 | Raw score for each item of the CBCL: 0 to 2 points |
| Internalizing Problems | T-score on the Internalizing scale of the CBCL |
| Externalizing Problems | T-score on the Externalizing scale of the CBCL |
| Internalizing-Externalizing Difference | The difference between T-scores on the Internalizing and Externalizing cales of the CBCL |
| Total T-score | T-score of the CBCL total score |
| T-score 1–T-score 8 | T-scores for each syndrome scale on the CBCL  T-score 1: Anxious/Depressed, T-score 2: Withdrawn/Depressed, T-score 3: Somatic Complaints, T-score 4: Social Problems, T-score 5: Thought Problems, T-score 6: Attention Problems, T-score 7: Rule-Breaking Behavior, T-score 8: Aggressive Behavior |
| CPSS Risk Percent | Risk (%) of developing SSD calculated by CPSS |
| Spring | Spring birth (March, April, May) |
| Summer | Summer birth (June, July, August) |
| Autumn | Autumn birth (September, October, November) |
| Winter | Winter birth (December, January, February) |
| Physical disorders | Physical disorders not due to a mental disorder (other than epilepsy) |
| Somatic symptom disorders | Somatic symptoms and Related Disorders (DSM-5) |
| Epilepsy | Epilepsy with EEG abnormalities |
| Neurodevelopment Disorder | Neurodevelopmental disorders (DSM-5) |
| Bipolar Disorder | Bipolar and Related Disorders (DSM-5) |
| Depressive Disorder | Depressive Disorders (DSM-5) |
| Anxiety Disorder | Anxiety Disorders (DSM-5) |
| Obsessive-Compulsive Disorder | Obsessive-Compulsive Disorders (DSM-5) |
| PTSD | Trauma and Stressor-Related Disorders (DSM-5) |
| Pharmacotherapy | With or without medication |
| Abuse | Abused/not abused by parents |
| Bullying Victimization | The extent of bullying: 0–3 |
| Social Withdrawal | Degree of social withdrawal: 0–3 |

CBCL, Child Behavior Checklist; Q, question/item number on the CBCL; T-score, standardized score with a mean of 50 and standard deviation of 10; CPSS, Clinical Prediction Score System; SSD, schizophrenia spectrum disorder; DSM-5, Diagnostic and Statistical Manual of Mental Disorders, Fifth Edition; EEG, electroencephalography; PTSD, post-traumatic stress disorder.

**3 Supplementary Appendix 1**

**The algorithm of the Child Psychosis-Risk Screening System (CPSS) using CBCL/6-18**

Risk indicators: p^a^, z^b^, risk % (or CPSS risk score)^c^

1. p=$\frac{1}{1+exp(-z)}$
2. z = −6.517+0.119×Tii-0.066×Tiii+0.031×Ti+0.035×Tiv+0.279×Tv+0.129×Tvi-0.011×Tvii-0.428×Tviii = logit(p)
3. p×100=risk %

Child Behavior Checklist (CBCL)/6-18 syndrome subscales

Ti: Anxious/Depressed

Tii: Withdrawn/Depressed

Tiii: Somatic Complaints

Tiv: Social Problems

Tv: Thought Problems

Tvi: Attention Problems

Tvii: Rule-Breaking Behavior

Tviii: Aggressive Behavior

For details, see reference 29.
